# Immunomodulatory Effects of High-Dose Irradiation Regimens in Renal Cell Carcinoma: Insights from an In Vitro Model with Human Peripheral Blood Mononuclear Cell

**DOI:** 10.3390/biomedicines13092107

**Published:** 2025-08-29

**Authors:** Fatima Meniai Merzouki, Guillaume Paul Grolez, Clément Bouchez, Bertrand Leroux, Jérôme Benoit, Olivier Morales, Nadira Delhem

**Affiliations:** 1 Inserm, CHU Lille, U1189-ONCO-THAI—Assisted Laser Therapy and Immunotherapy for Oncology, University of Lille, F-59000 Lille, France; guillaume.grolez@inserm.fr (G.P.G.); clement.bouchez@inserm.fr (C.B.); bertrand.leroux@inserm.fr (B.L.); olivier.morales@cnrs.fr (O.M.); 2 Departement of Radiotherapy, Centre Hospitalier de Beauvais, F-60000 Beauvais, France; 3 Oncovet, Platerforme PRECI, F-59650 Villeneuve d’Ascq, France; jerome.benoit@oncovet.fr; 4 CNRS, Inserm, CHU Lille, UMR9020-U1277-CANTHER—Cancer Heterogeneity Plasticity and Resistance to Therapies, University of Lille, F-59000 Lille, France

**Keywords:** renal cell carcinoma, high dose irradiation, immunophenotype, peripheral blood monocular cells, immunomodulation

## Abstract

**Background:** Stereotactic ablative radiotherapy (SABR) is increasingly used in the treatment of localized and metastatic renal cell carcinoma (RCC), a malignancy traditionally considered radioresistant. Beyond direct cytotoxicity, SABR may promote immunogenic cell death and modulate the tumor immune microenvironment, though the underlying mechanisms remain incompletely understood. **Objectives and Methods:** This study examined the immunomodulatory effects of two high-dose irradiation regimens (8 Gy and 3 × 8 Gy) in an in vitro model using two RCC cell lines (ACHN, Caki-2) and peripheral blood mononuclear cells (PBMCs) from healthy donors. **Results:** The 3 × 8 Gy regimen more effectively reduced tumor cell viability and proliferation, particularly in ACHN cells, suggesting differential radiosensitivity. Both regimens induced secretion of IL-6, IL-8, TGF-β, and VEGF, with levels varying by cell line and dose. Caki-2 cells exhibited a cytokine profile consistent with a pro-inflammatory and potentially immunosuppressive phenotype. Conditioned media from irradiated cells were used to stimulate PBMCs, revealing divergent responses. Media from 3 × 8 Gy-irradiated ACHN cells enhanced PBMC proliferation and increased CD8^+^ T cells and CD11c^+^ monocytes, along with IFN-γ, IL-2, and TNF-α secretion, suggesting immunostimulatory effects. Conversely, media from Caki-2 cells had minimal impact on PBMC proliferation and increased TGF-β levels. **Conclusions:** These results indicate that high-dose irradiation can differentially modulate immune responses in RCC cell lines, depending on tumor intrinsic properties and irradiation regimen. Further in vivo studies are warranted to validate these findings and support development of SABR immunotherapy combinations guided by predictive immune biomarkers.

## 1. Introduction

Renal cell carcinoma (RCC) comprises a heterogeneous group of kidney cancers, with clear cell RCC accounting for approximately 80% of cases. At diagnosis, around 15% of patients present with metastatic disease, and up to 20% develop metastases after treatment for localized tumors [1].The remaining cases correspond to non-clear cell RCC subtypes [2]. Despite advances in systemic therapies, including immunotherapy and anti-angiogenic agents, RCC remains a lethal malignancy with high rates of local recurrence and distant metastases, particularly in advanced stages.

RCC is traditionally considered radioresistant to conventional fractionated radiotherapy (2 Gy per fraction) [3]. Radiobiological studies have estimated the α/β ratio for RCC between 2.6 and 6.9, suggesting a potential benefit of delivering high-dose hypofractionated radiation to overcome this resistance [4]. Consequently, stereotactic ablative radiotherapy (SABR) has emerged as a promising technique that delivers ablative doses (>5 Gy per fraction) with high precision to small extracranial tumor volumes [5]. Technological advances have expanded SABR applications to patients with oligometastatic or oligoprogressive RCC [6], and early clinical studies indicate high local control rates with limited toxicity in small, inoperable renal tumors [7].

Beyond direct cytotoxicity, radiotherapy can modulate antitumor immunity. Clinical investigations combining radiotherapy and immunotherapy in RCC have reported encouraging responses, supporting the hypothesis that radiation enhances tumor immunogenicity and promotes immune-mediated tumor control [8]. In fact, SABR has been shown to induce the expression of tumor neoantigens and activate T lymphocytes by stimulating the production of type I interferon (INF-I) within the irradiated tumor [9]. Villafuerte et al., recently reviewed preclinical and pilot clinical data demonstrating that SABR induces immunomodulatory effects in RCC, including tumor neoantigen release, activation of T cells, and stimulation of type I interferon pathways, especially in cytoreductive contexts [10]. Importantly, lymphocytes critical mediators of antitumor immunity often decrease in number after conventional radiotherapy; however, SABR may reduce normal tissue irradiation and mitigate severe lymphopenia [11].

Although clinical trials such as FASTROCK II and IROCK have evaluated ablative regimens ranging from 26 Gy in a single fraction to 42 Gy over 3–10 fractions for primary RCC, these studies primarily focused on tumor control and tolerability without assessing immune-related endpoints [12,13]. Our objective was to investigate the immunomodulatory impact of high-dose irradiation on tumor–immune dynamics. The selected dose and fractionation schemes (1 × 8 Gy and 3 × 8 Gy) were based on prior preclinical evidence emphasizing radiobiological and immunological mechanisms, rather than mirroring clinical protocols. The 3 × 8 Gy regimen was chosen based on its demonstrated ability to activate the cGAS STING pathway and stimulate type I interferon responses key processes in initiating antitumor immunity [14,15,16]. In contrast, single high-dose irradiation (≥8 Gy) has been linked to suppression of STING activation via TREX1 upregulation [17], while still contributing to vascular damage and endothelial apoptosis mechanisms particularly relevant to RCC due to its pronounced vascularization [18,19]. The immunomodulatory effects of radiotherapy have been extensively characterized in other cancers, the specific mechanisms and consequences in RCC remain incompletely understood. The scarcity of tumor tissue available after radiotherapy limits translational studies. Investigating changes in the tumor microenvironment (TME) before and after irradiation, together with analyses of circulating immune cells, offers valuable insights into immune dynamics and biomarker identification. Despite these advances, there is a knowledge gap regarding how high-dose radiation influences peripheral blood mononuclear cell (PBMC) responses to factors secreted by irradiated RCC cells. We hypothesize that conditioned media from RCC cell lines exposed to high-dose irradiation modulates the PBMC phenotype and function in vitro. To test this, we evaluated the effects of conditioned media from two irradiated RCC cell lines on PBMCs isolated from healthy donors, focusing on immune cell proliferation and cytokine production. These data will provide mechanistic insights to inform the design of combined radiotherapy-immunotherapy approaches in RCC.

## 2. Materials and Methods

### 2.1. Cell Lines and Culture Conditions

Two human renal cell carcinoma (RCC) lines were used: ACHN (ATCC CRL-1611, Manassas, VA, USA), originally isolated from the pleural effusion of a 22-year-old male with metastatic renal adenocarcinoma, and Caki-2 (ATCC HTB-47, Manassas, VA, USA), derived from a primary clear cell carcinoma of a 69-year-old Caucasian male.

These lines differ in their Von Hippel–Lindau (VHL) gene status, with ACHN exhibiting wild-type VHL and Caki-2 harboring a mutated VHL genotype. ACHN cells were maintained in Eagle’s Minimum Essential Medium (Gibco, ThermoFisher, Waltham, MA, USA) supplemented with 10% fetal bovine serum (FBS) (Gibco, ThermoFisher, Waltham, MA, USA), 2 mM L-glutamine, 1% non-essential amino acids, and 100 µg/mL streptomycin. Caki-2 cells were cultured in McCoy’s 5A medium (Gibco, ThermoFisher, Waltham, MA, USA), containing 10% FBS, 2 mM glutamine, and 100 µg/mL streptomycin. Cells were incubated at 37 °C in a humidified atmosphere with 5% CO_2_. Culture supernatants were harvested at indicated time points, aliquoted, and stored at −80 °C without centrifugation or filtration to preserve soluble components.

### 2.2. In Vitro Irradiation Protocol

For irradiation experiments, 1 × 10^6^ cells were seeded in 75 cm^2^ culture flasks. After 24 h, the culture medium was refreshed, and flasks were placed between two polymethyl methacrylate (PMMA, USA) slabs to simulate tissue-equivalent scattering conditions, 4.5 cm beneath and 5 cm above the flask (Supplementary Figure S1). Cells were exposed to high-energy 6 MV photon beams using a clinical linear accelerator (TrueBeam^®^ LINAC, Varian, CA, USA) at 600 MU/min, delivering either a single dose of 8 Gy or three fractions of 8 Gy (3 × 8 Gy) administered at 24 h intervals. Control flasks were handled identically but not irradiated. The experimental timeline is described in the Supplementary Data Figure S2.

Dosimetric validation of dose delivery to the monolayer was performed by a medical physicist using Gafchromic™ film during mock irradiations to confirm uniform and precise exposure.

### 2.3. Colonogenic Assay

To evaluate the reproductive survival following irradiation, cells were plated at defined densities in 6-well plates and allowed to adhere. Subsequently, cells were irradiated with increasing doses (0, 2, 4, 6, and 8 Gy). Plates were incubated for 14 days, with medium replaced every 72 h. Colonies were fixed with methanol and stained with crystal violet (Santa Cruz Biotechnology, Dallas, TX, USA). Colonies containing ≥ 50 cells were quantified using ImageJ software [20] (version 1.54m 5 December 2024). Plating efficiency (PE) and surviving fraction were calculated as follows:

PE (%) = (colonies formed at 0 Gy/Number of cells seeded) × 100.

### 2.4. Cell Viability Assessment

Cell viability was determined using the CellTiter-Glo luminescent assay (Promega, Madison, WI, USA), which measures ATP as a surrogate for metabolically active cells according to the manufacturer’s instructions. RCC cells and peripheral blood mononuclear cells (PBMCs) were seeded at 1 × 10^5^ cells per well, in triplicate for each condition in white 96-well white Costar plates (Corning, Somerville, MA, USA). After experimental treatments, 100 µL of reagent was added per well and incubated at room temperature in the dark for 10 min. Luminescence was recorded using a ClarioStar Plus luminometer (BMG Labtech, Champigny sur Marne, France) driven by MARS™ Software v2.06. For PBMCs, cells were collected from healthy adult donors and were activated or not with plated anti-CD3 (1 μg/mL; Miltenyi, Bergisch Gladbach, Germany) and anti-CD28 (100 ng/mL; Clinisciences, Montrouge, France). Viability was measured after 3 days of co-culture with tumor-conditioned media (CM) collected 24 h post-irradiation (NT, 8 Gy, or 3 × 8 Gy). Data were expressed as relative luminescence units (RLU) or normalized to untreated controls. Normalized RLU = RLU of the sample/mean RLU of the untreated control.

### 2.5. PBMC Proliferation Assay

PBMCs were isolated by Ficoll density gradient centrifugation from whole blood collected from healthy donors using lymphocyte separation medium (Eurobio, Les Ullis, France) and leucosep 50 mL tubes (Eurobio, Les Ullis, France), under approved Institutional Review Board protocols at the Biology Institue of Lille (DC-2020-3942). Cells were cultured in ML10 medium made in Roswell Park Memorial Institute (RPMI) 1640 medium (Gibco, ThermoFisher, Waltham, MA, USA) supplemented with sodium pyruvate (1 mM) (Sigma-Aldrich, St. Louis, Mo, USA), MEM non-essential amino acids 1x (Sigma-Aldrich, St. Louis, Mo, USA), 25 mM HEPES (Sigma-Aldrich, St. Louis, Mo, USA), 50 µM 2-mercaptoethanol (Sigma-Aldrich, St. Louis, Mo, USA), 10 µg/mL gentamicin (Thermo Fisher Scientific, Waltham, MA, USA), and 10% Human Serum AB (SAB, Sigma-Aldrich, St. Louis, Mo, USA). PBMCs (1 × 10^5^ per well) were plated in round-bottom 96-well plates and stimulated with anti-CD3 (1 µg/mL) and anti-CD28 (100 ng/mL) or left unstimulated. PBMCs were cultured for 3 days with 50 μL of supernatant from each cancer cell line, which was collected 24 h post-irradiation under various conditions (non-treated, sn8 Gy, or sn3 × 8 Gy) and ML10: raw culture media of PBMCs), also called conditioned media, was added to PBMCs in 96 round-bottomed plates when stated. For proliferation analysis, cells were pulsed with [^3^H]-thymidine (1 µCi/well) for the final 18 h of a 3-day incubation. Incorporation was quantified by β-scintillation counting. At the end of the culture, the cells were harvested on a glass fiber filter (PerkinElmer, Courtaboeuf, France) using a Tomtec harvester (Wallac, Turku, Finland), then sealed in a sample bag (PerkinElmer, Courtaboeuf, France) with scintillation liquid (Beckman Coulter, Brea, CA, USA). Results were presented as counts per minute (CPM) and normalized to non-treated controls.

### 2.6. Flow Cytometry

Apoptosis and necrosis in irradiated RCC cells were assessed 24 h post-irradiation using an Annexin V-FITC/PI kit (Miltenyi Biotec, Bergisch Gladbach, Germany), and cells were analyzed on an Attune NxT cytometer (Thermo Fisher Scientific, Waltham, MA, USA). Cells were seeded as 2 × 10^6^ in a 6-well plate (Dutscher, Courtaboeuf, France) with 2 mL complete growth media. After overnight incubation, the cells were subjected to in vitro high dose irradiation, according to regimes as described above. The cells were trypsinized and recuperated 24 h post-irradiation, processed according to manufacturer’s instructions, and examined using Attune NxT (Thermo Fisher Scientific, Waltham, MA, USA) at the BioImaging Center Lille (BICeL) platform.

For PBMC analysis, cells cultured with CM for 72 h were stained with viability dye and fluorochrome-conjugated antibodies against surface and intracellular markers, including: anti-CD3-PECy7 mAb, anti-CD4 Vioblue, anti-CD8 Vio-green, anti-CD11c-APC, anti-CD335-APC, anti -CD14-PE, anti-CD25-FITC, anti-CD127-PE VIO770 and anti-FoxP3. All antibodies were provided from Miltenyi (Miltenyi Biotec, Bergisch Gladbach, Germany) (Supplementary Data Table S2). Fluorescence was analyzed by flow cytometry using the Attune NxT (Thermo Fisher Scientific, Waltham, MA, USA). Flow cytometry data were processed using FlowJo software (v10.8.1, Ashland, OR, USA), and the gating strategy is described in the Supplementary Materials Figure S3. The antibodies used for cytometry applied to individualize cell population is described in the Supplementary Materials Table S2.

### 2.7. Cytokine Quantification by ELISA

Quantification of vascular endothelial growth factor A (VEGF-A) in tumor cell supernatants was performed using a commercial human VEGFA ELISA kit (Cat No. ab119566; Abcam^®^ Biotech, Cambridge, UK), according to optimized assay protocols. Absorbance was measured at 450 nm using a ClarioStar microplate reader (Berthold Technologies, Oak Ridge, TN, USA).

For analysis of immune cytokines in co-culture experiments, PBMCs from healthy donors were incubated with conditioned media from RCC cells (collected 24 h after exposure to either 0 Gy, 8 Gy, or 3 × 8 Gy). After three days of culture, supernatants were harvested, and cytokines TNF-α, IFN-γ, IL-2, and IL-10 were quantified using a multiplex bead-based assay (Merck Millipore^®^, Darmstadt, Germany) on the Magpix^®^ platform (Luminex Corporation, Austin, TX, USA), following manufacturer-recommended procedures. Cytokine concentrations were calculated using standard curves generated for each target analyte.

Pro-inflammatory cytokines IL-6 and IL-8 in conditioned media from RCC and TGF-β levels in tumor conditioned media and PBMC supernatants were measured using an in-house sandwich ELISA. Briefly, 96-well plates (NUNC, Thermo Fisher Scientific, Waltham, MA, USA) were coated overnight at 4 °C with purified capture antibodies specific to human TGF-β (BD Pharmingen, San Jose, CA, USA). Plates were then washed with PBS-Tween 0.05% (PBS-T) (Sigma-Aldrich, St Louis, MO, USA) and a non-specific blocking with PBS-BSA 3% (Bovine Serum Albumin, Sigma-Aldrich, St. Louis, MO, USA) for 2 h at room temperature. Culture supernatants were added and incubated overnight at 4 °C. After washing, biotin-conjugated detection antibodies (1 μg/mL; BD Pharmingen ^TM^, San Jose, CA, USA), were applied for 90 min at room temperature. Signal amplification was achieved using streptavidin–HRP (1:10,000; Interchim, Montluçon, France), followed by development with an OPD/H_2_O_2_ substrate solution at 10 mg/ml (Sigma Aldrich, St. Louis, MO, USA). The enzymatic reaction was terminated with 2N HCl (VWR, Pennsylvania, USA), and absorbance was recorded at 492 nm using a CLARIOStar Plus microplate reader (BMG Labtech, Champigny-sur-Marne, France) controlled by MARS^TM^ software v2.06 (BMG Labtech, Champigny-sur-Marne, France).

Details of the antibodies used for quantification are provided in the Supplementary Materials Table S3.

### 2.8. Real-Time Quantification Polymerase Chain Reaction Assays (RTqPCR)

Total RNA was extracted from RCC cells (24 h post-irradiation) and PBMCs (after 3 days co-culture with CM) using Trizol reagent (Life Technologies, Carlsbad, CA, USA) method according to the manufacturer’s instructions. RNA purity and concentration were determined using a Nanodrop spectrophotometer (Thermo-Scientific, Thermosphere, Waltham, MA, USA). Reverse transcription was performed using 2 μg of total RNA. Then qPCR reaction was performed according to the manufacturer’s instructions, in a final volume of 20 μL, using 2X MESA GREEN qPCR MasterMix Plus for SYBR 258 Assay (Eurogentec, Seraing, Belgium) and the Mx3005PTM sequence detection system (Agilent technologies, Santa Clara, CA, USA). Transcripts were quantified using real-time quantitative RT-PCR with the Aria Mx system (Agilent technologies, Sasnta Clara, CA, USA). In PCR-96-LP-FLT plates (Corning Axygen, Corning, USA), 10 μL of a specific couple of primers (Eurogentec, Seraing, Belgium) and 1 μL of cDNA sample (equivalent to 10 ng of RNA/μL). The PCR program included initial denaturation for 5 min at 95 °C, followed by 40 standard amplification cycles as follows: 15 s at 95 °C (denaturation), then 1 min at 60 °C (annealing and elongation). Fluorescent products were detected at the last step of each cycle. The housekeeping genes: Glyceraldehyde-3-Phosphate Dehydrogenase (GAPDH), Hypoxanthine guanine PhosphoRibosyl Transferase (HPRT), and 18S RNA were used as controls. All primers were designed for real-time PCR (Table S2 in Supplementary) and purchased from (Eurogentec, Seraing, Belgium) or (Sigma–Aldrich, St. Louis, MO, USA).

Quantitative PCR was performed using SYBR Green Master Mix (Applied Biosystems) on the Agilent Aria Mx Real-Time PCR System.

Gene expression was quantified using the comparative cycle threshold method (2^−ΔΔCT^), as described by Livak and al [21]. Data were normalized to the expression of the housekeeping gene. For graphical representation, values were expressed as: 2^−ΔΔCT^ (reference condition set to 1) for bar plots, log_2_(2^−ΔΔCT^) (reference condition set to 0) for heatmaps. The value for each well was calculated using Agilent Aria Mx software (version 1.71). The reference of primers used for RTqPCR reaction exploring genes interest are provided in the Supplementary Materials Table S1.

### 2.9. Statistical Analysis

All results are expressed as means ± SEM of triplicates of at least three independent experiments. The normality’s distributions were assessed using the Shapiro–Wilk test. The statistical significance of differences between groups was determined by analysis of variance (ANOVA) followed by pairwise comparison using Tukey’s multiple comparisons tests for clonogenic assay. One-way ANOVA was used for impact of irradiation on PBMC viability, PBMC proliferation and cytokines release analysis. Due to the non-normality of data, the difference between proportion of PBMCs sub-population was assessed using the non-parametric Friedman test. All the significance levels were set to 0.05. All quoted *p*-values are two-sided. All data were analyzed using the statistical package GraphPad Prism 10.1 software (GraphPad, Software, San Diego, CA, USA).

## 3. Results

### 3.1. Direct Impact of High-Dose Irradiation on Cancer Cells

#### 3.1.1. High-Dose Irradiation Induced Distinct Effects on Radiosensitivity in RCC Cell Lines

The Cell capacity to form clones are essential in tumor growth. Cell survival curves were fitted using the Linear-Quadratic (LQ) model, which describes the relationship between radiation dose and surviving fraction according to the equation: SF = exp(−αD − βD^2^), where SF is the surviving fraction, D is the radiation dose, and α and β are constants representing the linear and quadratic components of cell killing, respectively [22].

**Figure 1:**
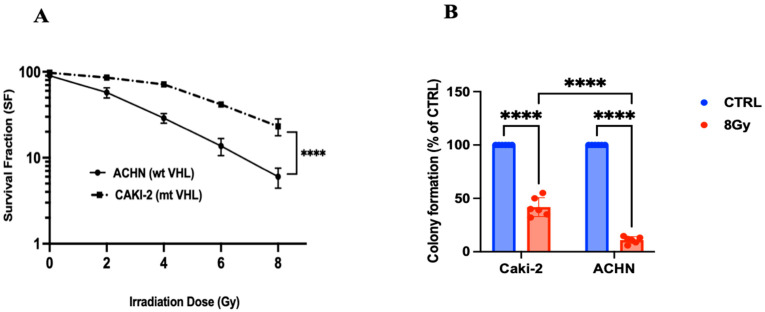
In Vitro clonogenic assay on ACHN and CAKI-2 cells line. (**A**) Dose–cell survival curves fitting by Linear-quadratic model in CAKI-2 and ACHN cells line. (**B**) Number of clones in both cell lines was quantified and normalized on control condition. Two-way ANOVA test was performed, and Tukey’s post hoc test was applied to correct for multiple comparisons (*n* = 6 per condition), *p* ≤ 0.0001 (****) being considered statistically significant or highly significant.

The results from our renal carcinoma cells line show a decrease in cell survival fraction starting at 4 Gy, with a more pronounced reduction in the ACHN cell line compared to the CAKI-2 cell line. We observed that this decrease in cell survival was even more significant at 8 Gy (Figure 1A), with an (88 ± 3%) reduction in capacity of the ACHN cell line to form clones, compared to a (51 ± 7%) reduction in the CAKI-2 cell line. These findings suggest that the ACHN cell line is more radiosensitive than the CAKI-2 cell line (Figure 1B).

#### 3.1.2. High-Dose Irradiation Reduced Cell Viability and Proliferation in Both Cell Lines

**Figure 2:**
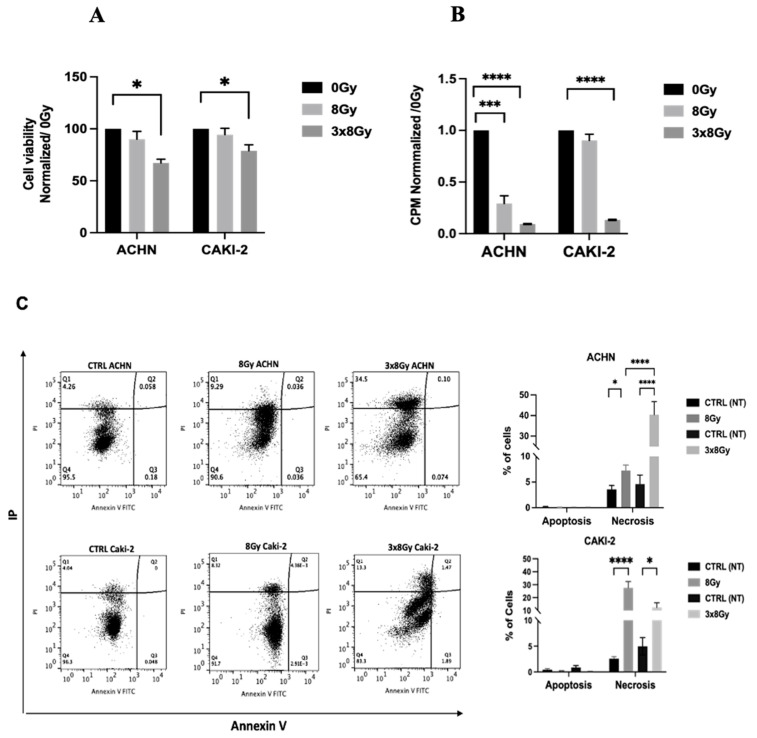
Impact of high dose irradiation on viability and proliferation of RCC cell lines, (**A**) cells viability, and (**B**) cells proliferation. The data are presented as normalized on control condition (0 Gy); results obtained 24 h after irradiation. Two-way ANOVA test was performed, Tukey’s post hoc test was applied to correct for multiple comparisons with *p* ≤ 0.05 (*), *p* ≤ 0.001 (***) and *p* ≤ 0.0001 (****) (*n* = 6 per dose group). (**C**) Analysis of mortality types in ACHN and CAKI-2 cell lines, 24 h post-irradiation using Annexin V-FITC/PI staining assay. At the right Annexin V/IP FACS plot in both cell lines. Histogram presentation at the left with the values expressed as a percentage of the parent population, where the values were compared with the non-treated. Two-way ANOVA test was performed (*n* = 5), with *p* ≤ 0.05 (*), *p* ≤ 0.001 (***), and *p* ≤ 0.0001 (****) being considered statistically significant or highly significant.

Cell viability was assessed 24 h after irradiation across both high-dose regimens. Following the 3 × 8 Gy regimen, both cancer cell lines exhibited a decrease in viability, with a more pronounced reduction in the ACHN line, which showed a viability rate of (67 ± 4%) compared to (79 ± 7%) for the Caki-2 cell line (Figure 2A). In contrast, the decrease in viability was not significant and remained similar for both cell lines after the 8 Gy regimen. Additionally, a significant reduction in proliferation was observed in both cell lines 24 h post-treatment with 3 × 8 Gy regimen compared to the control condition (Figure 2B). These results suggest that 3 × 8 Gy irradiation reduces metabolic activity and proliferation capacity of RCC cells in vitro. Cell death induced by high-dose irradiation was analyzed using an Annexin V/PI Assay. At 24 h post-irradiation, ACHN cells exposed to the 3 × 8 Gy protocol showed a marked increase in necrotic cells, reaching a necrosis rate of 42%, compared to 3.8% in the 0 Gy control and 7% in the 8 Gy condition (Figure 2C). These results suggest that high-dose irradiation enhances necrosis in ACHN cells in a dose-dependent manner. Conversely, the Caki-2 cell line exhibited a necrosis rate of 28% with the 8 Gy protocol, compared to 4% in the 0 Gy control and 12% with the 3 × 8 Gy protocol. Additionally, high-dose irradiation did not trigger apoptosis in either cell line according to this assay.

#### 3.1.3. High-Dose Irradiation Increased the Release of VEGF and Inflammatory Cytokines

The secretome is a key element of the tumor microenvironment. In this in vitro study, we conducted a quantitative analysis of pro-inflammatory cytokines such as IL-6 and IL-8, the immunosuppressive cytokine TGF-β, and VEGF-A production by two cell lines, measured 24 h post-irradiation. This analysis aimed to assess the effects of two irradiation protocols on cytokine secretion variations in the two lines. Results show that both cancer cell lines produce VEGF-A at baseline, with higher secretion levels in the Caki-2 line. Following irradiation with the 8 Gy protocol, VEGF release significantly increased in the Caki-2-line, averaging (720 ± 200 pg/mL), compared to (192 ± 58 pg/mL) in the ACHN line.

**Figure 3:**
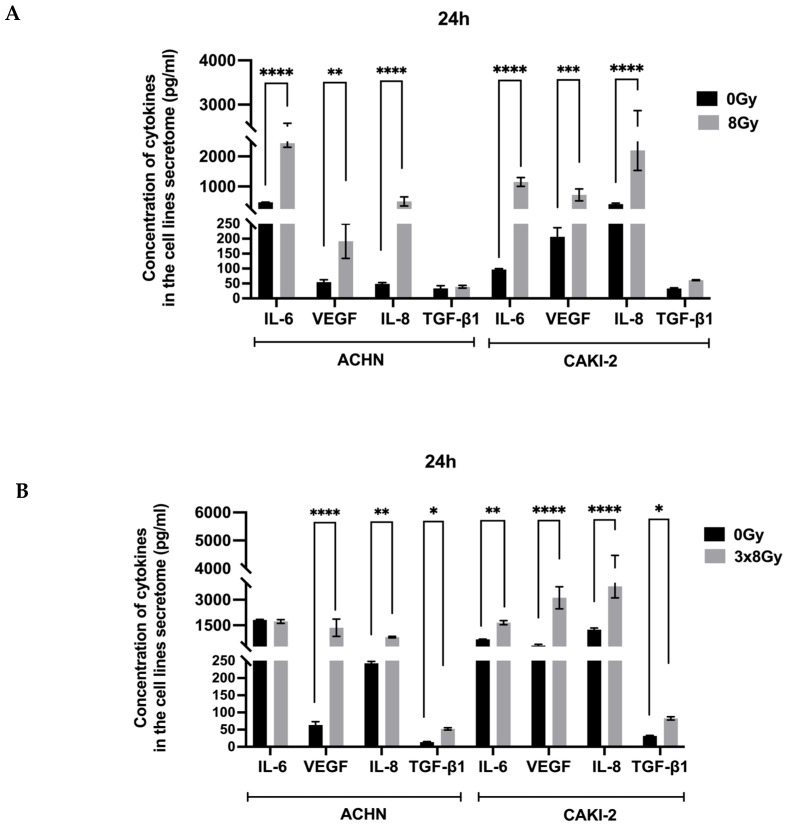
Dosage of inflammatory and immunosuppressive cytokines secretion by ELISA in cell lines supernatants collected 24 h post-irradiation. (**A**) Histogram showing the cytokine secretion after 8 Gy regimen. (**B**) Histogram showing the cytokine secretion after 3 × 8 Gy regimen. Results are expressed in cytokines concentration (pg/mL) and shown as mean ± SEM. Results are based on three independent experiments performed in triplicate. Statistical analysis was performed using Two-Way ANOVA, with normality confirmed through the Shapiro–Wilk test with *p* ≤ 0.05 (*), *p* ≤ 0.01 (**), *p* ≤ 0.001 (***), and *p* ≤ 0.0001 (****) being considered statistically significant or highly significant.

The 3 × 8 Gy regimen enhances VEGF-A secretion in both cell lines compared to controls, with a particularly marked increase in the CAKI-2 line’s conditioned media, where the concentration reaches (3116 ± 917 pg/mL) compared to (1356 ± 713 pg/mL) in the ACHN line. Similarly, both cancer cell lines secrete IL-6 at baseline, with notably higher levels in the ACHN line (474 ± 7 pg/mL) than in the Caki-2 line (97 ± 3 pg/mL). Following an 8 Gy single-dose irradiation, IL-6 secretion significantly rises in both lines, peaking at (2441 ± 137 pg/mL) for ACHN and (1150 ± 148 pg/mL) for Caki-2. The 3 × 8 Gy regimen also increases IL-6 production, though to a lesser extent than the 8 Gy protocol, with no significant difference from the control in ACHN cell line (1723 ± 184 pg/mL) with similar values in the control condition (1815 ± 59 pg/mL). These findings suggest that the 3 × 8 Gy regimen does not further elevate IL-6 secretion in the ACHN line, although the 8 Gy dose does. In contrast, IL-6 secretion in the Caki-2 line continues to increase under both irradiation conditions compared to the control. In terms of IL-8 release, the results show that both cancer cell lines secrete IL-8 at baseline levels, with higher concentrations observed in the Caki-2 line. Following a single fraction of 8 Gy, there is a significant increase in IL-8 secretion in the Caki-2 line, reaching a concentration of (2198 ± 667 pg/mL), while the ACHN line shows a lower concentration of (502 ± 150 pg/mL). The 3 × 8 Gy regimen leads to an even greater increase in IL-8 release from the Caki-2 line compared to control conditions. The ACHN line also shows a rise in IL-8 secretion after the 3 × 8 Gy treatment; however, this increase is considerably less pronounced than that seen in the Caki-2 line and does not significantly differ compared to the single 8 Gy treatment (Figure 3). In our experimental model of renal carcinoma, we observed an increase in TGF-β secretion in the supernatants of both cell lines 24 h after a single dose 8 Gy, despite baseline levels being similar. This increase was particularly observed in the Caki-2 line, which reached a concentration of (62 ± 2 pg/mL), compared to (52 ± 8 pg/mL) in the ACHN line. Following the 3 × 8 Gy irradiation, the Caki-2 line released even greater amounts of TGF-β, peaking at (83 ± 7 pg/mL) compared to (39 ± 5 pg/mL) for the ACHN line. Thus, while a single dose 8 Gy enhanced TGF-β secretion in both lines, the 3 × 8 Gy regimen significantly boosts TGF-β production in the Caki-2 supernatants compared to the ACHN line.

#### 3.1.4. High-Dose Effect on Transcriptomic Expression of PD-L1 and Inflammatory Cytokines

**Figure 4:**
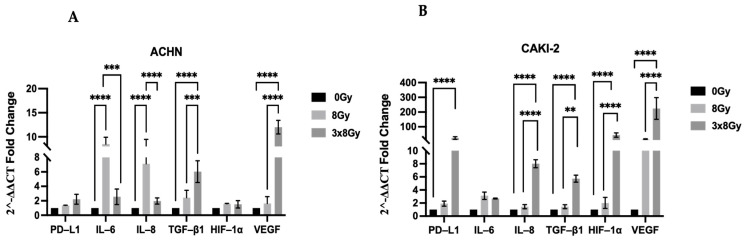
Differential expression of PD-L1, VEGF, HIF-1α, and inflammation-associated cytokine genes 24 h post-irradiation in both RCC cell lines, comparing irradiated versus non-irradiated controls: (**A**) ACHN cell line and (**B**) CAKI-2 cell line. Histograms show the 2^−ΔΔCT^ expression levels of the genes of interest. Data represent four independent experiments performed in triplicate, expressed as means ± SEM (*n* = 4) and normalized to each cell line’s untreated control condition (0 Gy). Statistical differences were analyzed using a non-parametric Mann–Whitney multiple comparison test. ** *p* < 0.01, *** *p* < 0.001, **** *p* < 0.0001.

To further characterize the phenotypic changes in cells after irradiation, we analyzed the gene expression of growth factor VEGF, HIF-1α, and cytokines involved in the inflammatory response in our two cancer cell lines. Overall, we observed an increase in pro-inflammatory cytokine transcripts, with variations based on dose and cell line. Notably, HIF-1α and VEGF transcripts were significantly elevated after 3 × 8 Gy irradiation in the CAKI-2 line, along with increases in IL-8 and TGF-β transcripts. However, the ACHN line showed a decrease in IL-8 and IL-6 expression after a 3 × 8 Gy regimen, but interestingly, TGF-β expression increased despite the absence of a corresponding increase in the concentration measured by ELISA. These results are generally in agreement with the quantification of cytokines carried out by ELISA. Furthermore, the 3 × 8 Gy protocol significantly upregulated PD-L1 expression in the CAKI-2 line, whereas the ACHN line did not show this increase under the same treatment conditions (Figure 4).

#### 3.1.5. Influence of High Dose Irradiation on the Induction of Type I Interferon and c-Gas/Sting Pathway

**Figure 5:**
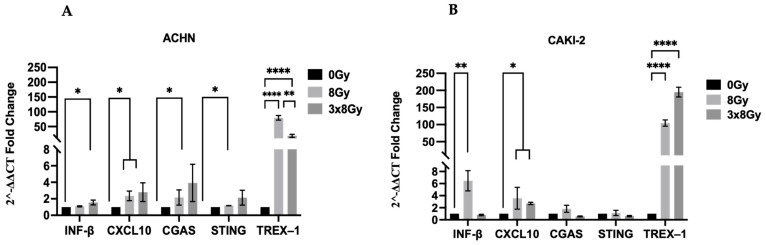
Differential expression of genes associated with type I interferon and c-GAS/Sting signaling pathway 24 h post-irradiation in both cell lines, comparing irradiated versus non-irradiated controls: (**A**) ACHN cell line and (**B**) Caki-2 cell line. Histograms show the 2^−ΔΔCT^ expression levels of the genes of interest. Data represent four independent experiments performed in triplicate, expressed as means ± SEM (*n* = 4) and normalized to each cell line’s untreated control condition (0 Gy). Statistical differences were analyzed using a non-parametric Mann–Whitney multiple comparison test. * *p* < 0.05, ** *p* < 0.01, **** *p* < 0.0001.

In our experimental model, in addition to secretome analysis, we assessed dose-dependent variations in gene expression 24 h post-treatment using RT-qPCR. The investigation targeted genes associated with the cGAS/STING signaling pathway, and its antagonist (TREX-1), and the type I interferon signaling pathway (CXCL-10, IFN-β). Both high-dose irradiation protocols significantly increased the expression of IFN-β and TREX-1 transcripts in both cell lines compared to the control group. However, TREX-1 expression was more pronounced in CAKI-2 under both irradiation regimens, while its expression decreased following 3 × 8 Gy treatment in the ACHN cell line. Additionally, an increase in CXCL-10 chemokine expression was observed. These results highlight the effect of high-dose irradiation on activating the type I interferon pathway in our RCC model (Figure 5).

Moreover, high-dose irradiation had differential effects on the cGAS/STING signaling pathway transcript levels in the two cancer cell lines. Notably, following 3 × 8 Gy irradiation, the ACHN cell line exhibited increased cGAS and STING expression along with reduced TREX-1 expression compared to CAKI-2.

### 3.2. Indirect Impact of High-Dose Irradiation on PBMCs

#### 3.2.1. Effect of RCC’s Lines Irradiated Supernatants on PBMCS Viability and Proliferation

**Figure 6:**
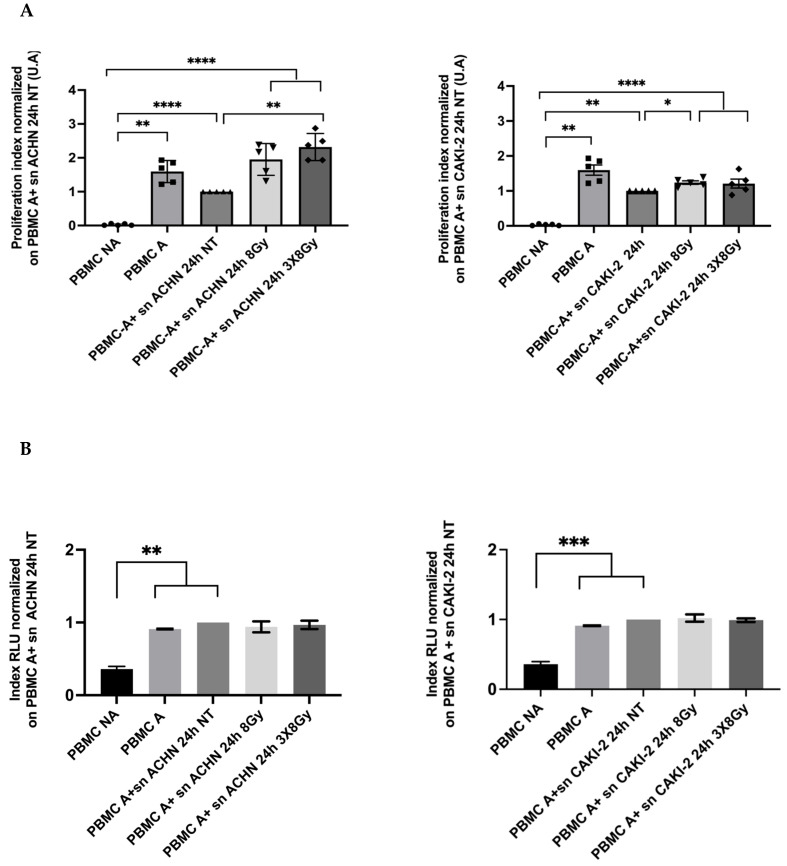
(**A**) Proliferation assay of activated PBMCs from five healthy donors treated with supernatants from ACHN and Caki-2 recovered 24 h after irradiation. Proliferation data are shown in counts per minute (CPM) and are normalized against the untreated condition. (**B**) Viability assay of activated PBMCs treated with supernatants from ACHN and Caki-2 recovered 24 h after irradiation. Viability data are presented in relative light units (RLU), also normalized to the untreated control (NT). PBMC A: ML10 culture media of PBMC, sn NT: supernatants of non-treated condition, sn 8 Gy: supernatant of treated condition 8 Gy, sun 3 × 8 Gy: supernatant of treated condition 3 × 8 Gy. Statistical differences were performed using One-way ANOVA, Tukey’s post hoc test was applied to correct for multiple comparisons with *p* ≤ 0.05 (*), *p* ≤ 0.01 (**), *p* ≤ 0.001 (***), and *p* ≤ 0.0001 (****), where *p* ≤ 0.05 is considered statistically significant and the others highly significant (*n* = 5).

To assess the impact of the RCC cell line secretome on the viability and proliferation of human PBMCs, we cultured PBMCs from five different healthy donors with supernatants collected from RCC cells 24 h post-irradiation or untreated. Figure 6 shows that the supernatants of the ACHN line in the two irradiation regimes 3 × 8 Gy and 1 × 8 Gy significantly improved the normalized proliferation of activated human PBMCs after 72 h of culture. This increase in proliferation was approximately two-fold greater in the treated conditions than in the untreated control. For the Caki-2 cell line, however, no increase in PBMC proliferation was observed under the 3 × 8 Gy treatment. Additionally, irradiated supernatants from both cell lines did not impact the viability of activated PBMCs.

#### 3.2.2. Impact of Irradiated Supernatants from RCC Cell Lines on Cytokine Release in Circulating Immune Cells

Conditioned media collected 24 h after irradiation of ACHN cells stimulated increased proliferation in PBMC cultures. Based on this observation, we further analyzed the phenotype and cytokine secretion profile of PBMCs, focusing on both immunostimulatory cytokines (IFN-γ, IL-2, TNF-α) and immunosuppressive cytokines (IL-10 and TGF-β).

Exposure to irradiated ACHN supernatants, from both single-dose (8 Gy) and fractionated (3 × 8 Gy) regimens, led to elevated secretion of IFN-γ and TNF-α in activated PBMCs compared to those cultured with non-irradiated ACHN supernatants. IL-2 levels were also notably higher in PBMCs exposed to supernatants from the 3 × 8 Gy-irradiated ACHN cells, indicating an enhanced pro-inflammatory response.

**Figure 7:**
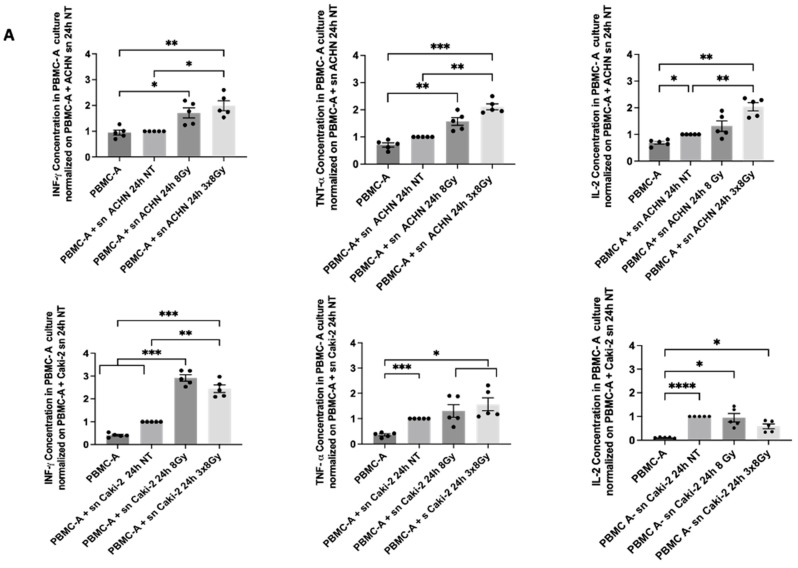

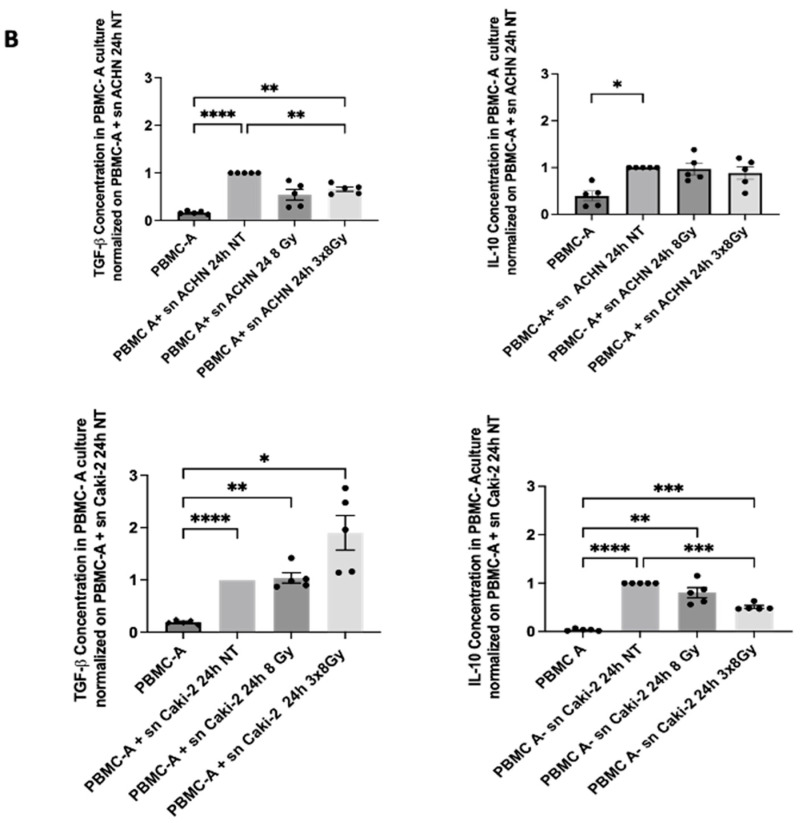
Quantification of pro-inflammatory and immunosuppressive cytokine secretion by activated PBMCs cultured with conditioned media from irradiated or non-irradiated RCC cell lines. Cytokine concentrations were measured by multiplex ELISA in the supernatants of activated peripheral blood mononuclear cells (PBMC-A) from healthy donors cultured for 72 h with conditioned media from ACHN and Caki-2 cells, collected 24 h after exposure to irradiation or control conditions. Data are normalized to cytokine levels in activated PBMCs cultured with tumor conditioned media not treated (PBMC-A—sn NT) and are presented as mean ± SEM. Results represent three independent experiments. (**A**): Pro-inflammatory Cytokine secretion following PBMC-A culture with ACHN irradiated or not supernatants and Caki-2 irradiated or not supernatants. (**B**): Immunosuppressive Cytokine secretion following PBMC-A culture with ACHN irradiated or not supernatants and Caki-2 irradiated or not supernatants. Results are presented as means ± SEM (*n* = 5). Statistical differences between conditions were assessed using a One-way ANOVA test, Tukey’s post hoc test was applied to correct for multiple comparisons, with significance levels set at *p* ≤ 0.05 (*), *p* ≤ 0.01 (**), *p* ≤ 0.001 (***), and *p* ≤ 0.0001 (****), where *p* ≤ 0.05 is considered statistically significant and the others highly significant. (sn = supernatants).

In contrast, supernatants derived from CAKI-2 cells irradiated with the 3 × 8 Gy regimen induced a reduction in IL-2 secretion in PBMC cultures relative to the non-irradiated condition. While IFN-γ levels appeared to increase following exposure to CAKI-2 supernatants irradiated with 8 Gy, TNF-α secretion did not show a marked change across conditions (Figure 7A).

Regarding immunosuppressive cytokines, PBMCs cultured with ACHN 3 × 8 Gy supernatants exhibited a decrease in TGF-β secretion compared to those treated with non-irradiated supernatants. Conversely, treatment with CAKI-2 supernatants exposed to the 3 × 8 Gy regimen led to an increase in TGF-β production (Figure 7B). IL-10 levels were generally reduced in PBMCs treated with irradiated CAKI-2 supernatants exposed to the 3 × 8 Gy regimen, while they remained stable in cultures treated with ACHN-derived supernatants, regardless of irradiation status.

Taken together, these findings suggest that irradiation of ACHN cells enhances the immunostimulatory potential of their secretome, promoting a pro-inflammatory cytokine profile in PBMCs. In contrast, CAKI-2 supernatants, particularly after fractionated high-dose irradiation, may favor an immunosuppressive milieu characterized by increased TGF-β secretion and reduced IL-2 production. These differences may underline the contrasting effects on PBMC proliferation observed between the two cell line conditions.

#### 3.2.3. High Dose Irradiation Induced Immunomodulation in Circulating Immune Cells in RCC Models

**Figure 8:**
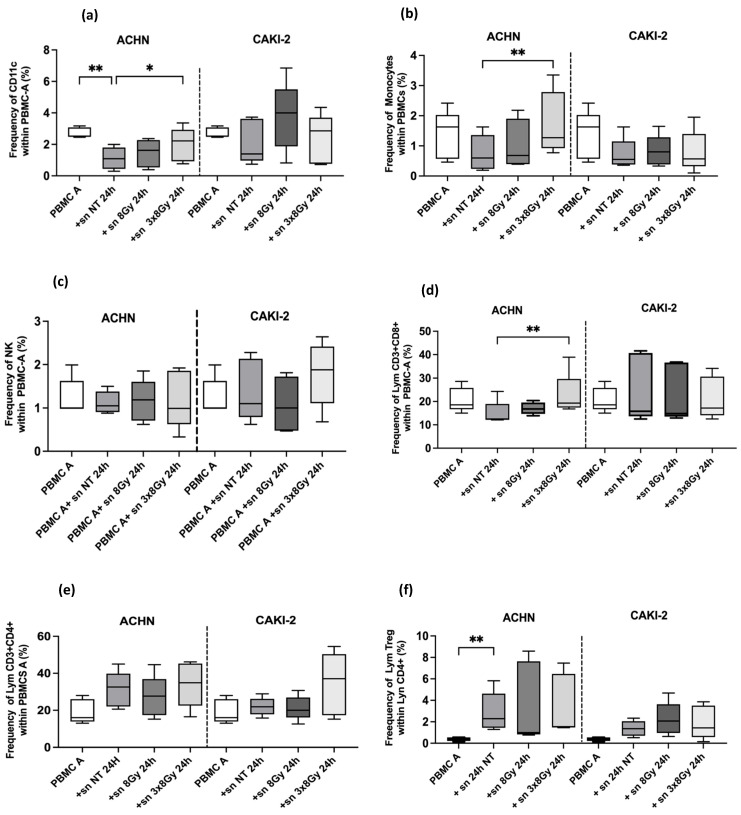
The immune phenotype of PBMCs from five healthy donors was assessed following activated PBMC culture with conditioned media from ACHN and CAKI-2 cell lines, both treated and untreated. The data are presented as percentages of total PBMC, except for Treg T cells, which are expressed as a percentage of CD4+ T cells. The medians are represented by the midlines of box-and-whisker plots, with the boxes indicating the 25th and 75th percentiles, and the whiskers representing the minimum and maximum values for the compared groups. The non-parametric Friedman test was employed, with asterisks indicating the statistical significance of observed differences at *p* ≤ 0.05 (*) and *p* ≤ 0.01 (**). Panels (**a**–**c**) show a comparative representation of innate immune populations, while panels (**d**–**f**) illustrate a comparative representation of adaptive immune populations and T regulatory cells (sn = supernatants).

For a better understanding of PBMC responses to high-dose irradiation through the supernatants of the irradiated cell lines, the proportions of peripheral cell subsets in healthy donors were compared following culture with supernatants from RCC cell lines, treated or untreated. Regarding innate immune cells. The dendritic cells were identified by surface expressions CD11c and lack of CD3. The proportion of CD11c was significantly higher in the ACHN-treated supernatant at 3 × 8 Gy compared to the untreated condition ((2 ± 1%) versus (1.11 ± 0.70%), with *p* = 0.03). Conversely, a decrease in the frequency of CD11c+ cells was noted after culturing PBMCs with untreated ACHN supernatants compared to the activated PBMCs from healthy donors ((1.11 ± 0.70%) versus (3 ± 0.33%), with *p* = 0.052)). However, there was no difference in proportion of dendritic cells and monocytes in all Caki-2 supernatants treated (Figure 8a). The monocytes proportion cells were identified by surface expressions CD14 and lack of CD3. The proportion of CD14 was significantly higher in the ACHN-treated supernatant at 3 × 8 Gy compared to the untreated condition ((1.73 ± 1.05%) versus (0.76 ± 0.6%), with *p* = 0.009) (Figure 8b). The NK cells proportion were identified by surface expressions CD335 and lack of CD3. There was no difference in proportion of NK cells after culturing with supernatant treated conditions in both cells line. Regarding adaptive immune populations, lymphocytes expressing both CD3 and CD8 were gated as CD8+ T cells, while those expressing both CD3 and CD4 were gated as CD4+ T cells. The proportion of CD8+ T was significantly increased in the ACHN-treated supernatant at 3 × 8 Gy compared to the untreated condition ((23 ± 9%) versus (15 ± 5.2%), with *p* = 0.007) (Figure 8d). It is also noted that untreated tumor supernatants induced a decrease in the proportion of CD8+ T cells (15 ± 5.2%) compared to healthy donors ((21 ± 5.25%), with *p* = 0.052). No differences in CD 8+ T proportion were observed across all CAKI-2 treated supernatants. Additionally, the proportion of CD4+ T cells increased in the 3 × 8 Gy supernatant treated condition (35 ± 17%) compared to the untreated conditions in the CAKI-2 supernatant 3 × 8 Gy condition (22 ± 5%), though not statistically significant, likely due to limited number of healthy donors in this study. The Tregs proportion was identified is by surface expressions of both CD4 and CD25 and the absence of CD127. Flow cytometry analysis demonstrated that treatment with conditioned media from irradiated RCC cell lines did not significantly affect the frequency of regulatory T cells in PBMC cultures. Specifically, no significant differences were observed between treated and control conditions for supernatants derived from CAKI-2 cells (8 Gy: *p* = 0.60; 3 × 8 Gy: *p* = 0.17) and ACHN cells (8 Gy: *p* = 0.48; 3 × 8 Gy: *p* = 0.84). The gating strategy of different sub-populations is shown in supplementary data.

#### 3.2.4. Influence of Irradiated Supernatants from RCC Cells on Human PBMCs Genes Expression

**Figure 9:**
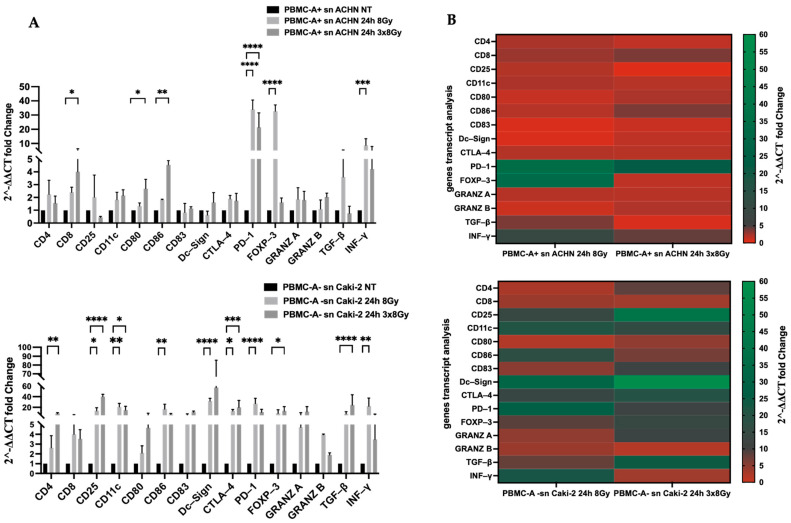
Gene relative expression analysis of activated PBMCs from healthy donors cultivated with conditioned media of ACHN and Caki-2 cells collected 24 h post-irradiation and analyzed at 72 h. (**A**) Histogram representation of the evolution of relative gene expression shows levels of genes associated with inflammation, maturation properties of dendritic cells, and immune checkpoints. Data, representative of five donors, were normalized to gene expression in the untreated condition (NT). Statistical differences were assessed using Two-Way ANOVA test or a Kruskal–Wallis test. * *p* < 0.05, ** *p* < 0.01, *** *p* < 0.001, **** *p* < 0.0001. (**B**) Heatmaps, expressed in 2^−ΔΔCT^ normalized to the untreated condition, illustrate the differential expression of 15 genes in PBMCs. Squares in light green denote genes largely overexpressed (30 folds higher compared with the untreated condition), squares in dark green represent genes overexpressed (over 20 folds compared with the untreated condition), squares in dark red indicate genes overexpressed between 5 and 10 folds, and squares in light red indicate under-expressed genes (sn = supernatants).

In our experimental model using conditioned culture media, we observed that radiation-induced signals from irradiated renal carcinoma cell lines modulated the secretome and transcription of inflammatory mediator genes in the cell lines. To further study the effect on PBMCs, gene expression was analyzed by RT-qPCR. All results were normalized to those obtained under untreated conditions. Figure 9A displays the relative results, while Figure 9B provides a summary in a more understandable format. We observed upregulation of the INF-γ gene in the treated conditions, with a more pronounced effect of supernatants treated with a single dose of 8 Gy in both cell lines. Notably, there was a significant increase in TGF-β transcript expression following culture with supernatants treated with 3 × 8 Gy. In contrast, TGF-β gene expression decreased after culturing with ACHN line supernatants under the same conditions, although this change was not statistically significant compared to the untreated conditions. Interestingly, we observed an increase in the expression of dendritic cell maturation markers, including CD80, CD86, and DC-Sign, particularly after culture with the CAKI-2-line supernatants, regardless of the dose. Additionally, supernatants from the CAKI-2 cell line induced a significant upregulation of CD4, CD25, and FOXP3 gene expression. It is important to note that conditioned media from both cell lines led to elevated PD-1 transcript expression, while CTLA-4 expression was significantly higher in supernatants treated from the CAKI-2 line, regardless of the irradiation regimen.

## 4. Discussion

The evolving understanding of radiotherapy has underscored its dual role in achieving local tumor control and modulating antitumor immunity. In particular, the fractionation and timing of radiation delivery are now recognized as critical parameters influencing both therapeutic efficacy and immune activation, yet they remain incompletely defined in the context of renal cell carcinoma (RCC). Stereotactic ablative body radiotherapy (SABR), enabled by recent technological advancements, delivers high dose hypo fractionated radiation with sub millimetric precision and has demonstrated strong local control in RCC metastases and primary tumors deemed inoperable [8,23].

Traditionally considered radioresistant due to its limited response to conventional fractionation (2 Gy per fraction), RCC has shown renewed radiosensitivity under high-dose regimens (>5 Gy/fraction), as supported by radiobiological modeling and emerging preclinical evidence. Beyond direct cytotoxicity, high-dose irradiation can stimulate antitumor immunity by inducing immunogenic cell death, promoting the release of damage-associated molecular patterns (DAMPs), and enhancing tumor antigen presentation—features central to the concept of SABR acting as an in situ vaccine [24,25]. However, these effects appear to depend on the radiation dose and schedule: while fractionated regimens such as 3 × 8 Gy have been associated with enhanced activation of the cGAS-STING pathway and type I interferon signaling, single high doses (≥8 Gy) may trigger TREX1-mediated degradation of cytosolic DNA, thereby dampening immune activation [14].

In this context, our study aimed to address a critical gap: how high-dose irradiated RCC cells influence immune cell behavior through the release of soluble factors. Unlike most existing SABR studies, which focus on tumor control or safety endpoints [26], we investigated the immunomodulatory potential of irradiated RCC cell secretome on peripheral blood mononuclear cells (PBMCs), evaluating cytokine production and lymphocyte proliferation in vitro. While our model does not replicate the complexity of the tumor microenvironment, lymphatic architecture, or systemic immune circulation, it provides preliminary insight into radiation-induced immune modulation and highlights the relevance of tumor heterogeneity in shaping immune responses.

Moreover, the rarity of the abscopal effect, a phenomenon where localized irradiation induces regression in distant non-irradiated tumors, underscores the need to better understand the conditions under which SABR enhances systemic immunity particularly in RCC, where such events have been observed but remain mechanistically elusive [27]. Given the absence of a universally accepted SABR regimen for RCC, our findings underscore the importance of preclinical models that dissect the immunological consequences of different fractionation strategies to guide rational combinations with immunotherapies.

Despite these advances, investigations on how high-dose irradiation affects the systemic immune response and modulates the PBMC repertoire are scarce, particularly in the context of RCC treated with SABR. A preclinical study delivering 48 Gy in three fractions to human RCC xenografts in mice demonstrated effective local tumor control [28], yet no universally standardized SABR protocol exists. For instance, single-dose irradiation of 26 Gy is frequently used for small primary tumors (<4 cm), whereas larger tumors are treated with 42 Gy in three fractions of 14 Gy, aiming for a biologically effective dose (BED) of 100 Gy to optimize tumor control [29].

Though preclinical data on SABR-induced immune activation are limited, emerging evidence supports that a 3 × 8 Gy regimen more effectively stimulates the immune system compared to a single high-dose regimen. This immune activation is linked to the type I interferon signaling pathway via cGAS/STING, which is inhibited by the exonuclease TREX1 after exposure to a single 20 Gy dose [14]. Radiation-induced immune modulation in peripheral blood is further characterized by increased immunosuppressive cells, altered Th1/Th2 balance, and impaired effector T cell and dendritic cell (DC) functions, all of which may impact treatment response and prognosis.

In this study, we assessed immune modulation in human PBMCs from healthy donors using an in vitro RCC cell line model subjected to high-dose irradiation. It is well established that irradiated tumor cells secrete inflammatory cytokines and chemokines capable of activating the innate immune system. We examined the effects of irradiated supernatants from two RCC cell lines (CAKI-2 and ACHN) exposed to 8 Gy and 3 × 8 Gy regimens on PBMC phenotype and function.

Our results demonstrate that the 3 × 8 Gy regimen significantly reduced viability and proliferation in both RCC cell lines, with a more pronounced effect observed in ACHN cells. These findings suggest that a higher cumulative dose is associated with reduced tumor cell viability, indicating a potential to overcome radioresistance in RCC and suggesting enhanced efficacy. Cell death analysis revealed a dose-dependent increase in necrosis, with the 3 × 8 Gy regimen inducing greater necrosis in ACHN cells, whereas 8 Gy was more effective in CAKI-2 cells. The elevated necrosis in CAKI-2 after single-dose irradiation may reflect reduced DNA repair capacity or deficient cell-cycle checkpoint activation. Conversely, increased necrosis in ACHN under fractionated irradiation likely results from cumulative DNA damage or a failure to mount adaptive responses. These differences underscore intrinsic heterogeneity among RCC subtypes, particularly in DNA repair mechanisms and checkpoint activation [30].

Radiation-induced DNA damage can lead to mitotic catastrophe, characterized by aberrant mitosis and subsequent cell death, often through necrosis [31]. Necrosis, especially when triggered by mitochondrial reactive oxygen species (ROS) [32], is highly immunogenic due to the release of DAMPs, which activate immune responses [33]. This inflammatory cell death may contribute to immunogenic effects, although such effects were not directly assessed in this study.

Cytokine analysis revealed that high-dose irradiation increased VEGF secretion, particularly in CAKI-2 cells treated with the 3 × 8 Gy regimen. This cell line-specific VEGF production is probably influenced by VHL status; ACHN cells harbor wild-type VHL (VHL+/+) and secrete lower VEGF levels compared to CAKI-2 cells with mutated VHL (VHL−/−), consistent with prior reports [34]. Although hypoxia is a well-known driver of VEGF in vivo, our in vitro data indicate that radiation alone can modulate VEGF secretion independently of vascular dynamics. VEGF is known to promote angiogenesis and has been implicated in tumor radioresistance and immune evasion, although these effects were not directly assessed in our study.

Additionally, high-dose irradiation enhanced IL-6 release in both cell lines. ACHN cells constitutively produce IL-6 and soluble IL-6 receptor (sIL-6R), which may potentiate signaling through a paracrine feedback loop [26,29]. Increased IL-8 levels were also observed post-irradiation, especially in CAKI-2 supernatants treated with 3 × 8 Gy, supporting IL-8’s role in radiation-induced inflammation [35]. Elevated IL-8 serum levels correlate with poor response to anti-angiogenic and immunotherapy in metastatic RCC, suggesting IL-8 as a potential biomarker for treatment outcomes [36].

In the ACHN model, we observed a discrepancy between TGF-β mRNA and protein levels, which may be attributed to multiple layers of cytokine regulation. This lack of concordance likely reflects post-transcriptional control mechanisms or delays in protein translation and secretion, both of which are well-documented features of cytokine biology [37]. Moreover, TGF-β is secreted as a latent, inactive complex that requires extracellular activation to become functionally detectable, complicating direct protein measurement in vitro [38]. These regulatory complexities may account for the absence of detectable increases in bioactive TGF-β protein despite elevated transcript levels within the timeframe of our experimental conditions.

We further demonstrated that high-dose irradiation activates the type I interferon pathway, evidenced by increased IFN-β and TREX1 expression and upregulation of PD-L1, particularly in CAKI-2 cells at 3 × 8 Gy. The induction of PD-L1 via the cGAS-STING-IFN pathway has been previously described in irradiated tumors [33,34], linking DNA damage to immune modulation.

The RCC secretome, enriched in DAMPs and pro-inflammatory cytokines, plays a pivotal role in shaping the tumor microenvironment. These factors drive DC maturation, thereby initiating adaptive immune responses. However, aberrant DC activation may lead to immune tolerance. In our study, supernatants from 3 × 8 Gy-irradiated ACHN cells increased the proportion of DCs (CD11c+) and monocytes (CD14+), indicating enhanced innate immune activation. Notably, PBMCs exposed to these supernatants exhibited an increased frequency of CD8+ T cells, accompanied by elevated IFN-γ and TNF-α and decreased TGF-β levels, consistent with a TH1-type anti-tumor immune response.

In contrast, supernatants from CAKI-2 cells induced immunosuppressive effects, likely related to their radio resistance and high VEGF secretion. The observed DC maturation profile suggests a semi-mature or tolerogenic state rather than full maturation. This aligns with previous findings that VEGF impairs DC function by promoting a partial maturation phenotype characterized by expression of co-stimulatory molecules such as CD80 and CD86 but lacking effective T cell stimulatory capacity [39].

Interestingly, PBMC cultures treated with supernatants from irradiated CAKI-2 cells showed a significant upregulation of CD4, CD25, and FOXP3 mRNA expression (*p* < 0.05). In contrast, flow cytometry did not demonstrate a significant difference in the frequency of regulatory T cells among the treatment groups. This discrepancy may reflect transient FOXP3 expression in activated CD4+ T cells rather than stable Treg differentiation. Prolonged culture or additional factors may be required to consolidate the Treg phenotype [40].

The upregulation of PD-1, CTLA-4, and FOXP3 transcripts in PBMCs suggests possible immune checkpoint activation, but protein-level validation and functional assays are necessary to confirm immunosuppressive effects.

Moreover, PBMC exposure to untreated tumor supernatants increased TNF-α secretion, consistent with observations in RCC patients [41]. RCC is characterized by systemic immune dysregulation, including elevated IL-10, TGF-β, and Tregs, which collectively contribute to immune suppression [42]. Radiotherapy has been shown to enhance immune infiltration, antigen expression, and induce immunogenic cell death [43,44,45,46] and ongoing clinical trials are investigating combined radiotherapy and immunotherapy approaches. However, predictive biomarkers for treatment response remain limited due to scarce post-treatment tumor and immune profiling data.

This study provides evidence that high-dose irradiation can modulate the peripheral immune response in an in vitro model of renal cell carcinoma (RCC) through distinct cellular and molecular mechanisms, supporting its potential as an immunomodulatory strategy in RCC treatment. The immune responses differed substantially between the two RCC cell lines, ACHN and Caki-2, reflecting intrinsic differences in radiosensitivity, cytokine secretion patterns, and interindividual variability in the responsiveness of peripheral blood mononuclear cells.

Specifically, the radiosensitive ACHN cells promoted an immunostimulatory environment, characterized by enhanced PBMC proliferation and increased secretion of pro-inflammatory cytokines. In contrast, Caki-2 cells, which displayed features of radio resistance, produced a predominantly immunosuppressive secretome, marked by elevated levels of IL-6, IL-8, VEGF, and TGF-β. These divergent responses are consistent with the hypothesis that tumor intrinsic properties may influence radiation-induced immune modulation. This better reflects the correlative nature of our observations.

## 5. Conclusions

Taken together, these findings highlight the complexity of immune modulation induced by high-dose irradiation in an in vitro setting and emphasize the importance of considering tumor heterogeneity when developing combinatorial radiotherapy and immunotherapy strategies.

Our model provides preliminary evidence of how irradiated RCC cells may influence PBMC behavior, suggesting potential immunomodulatory mechanisms that require further functional validation. It lacks the full physiological context of a systemic immune network, including circulation, lymphoid structures, and the tumor microenvironment. Therefore, future in vivo studies and clinical investigations are essential to validate these observations and to further delineate the interactions between irradiated tumor cells and the immune system. Such work will be critical for identifying predictive immune biomarkers, refining patient stratification, and optimizing the efficacy of combined treatment regimens for renal cell carcinoma.

## Data Availability

The data that support the findings of this study are available from the corresponding author upon reasonable request.

## Supplementary Materials

The following supporting information can be downloaded at: https://www.mdpi.com/article/10.3390/biomedicines13092107/s1, Figure S1: Overview of the dosimetry used for irradiation of ACHN & Caki-2 cells in-vitro; Figure S2: Experimental timeline; Figure S3: strategy gating for characterizing the different subpopulations among PBMCs; Table S1: References of primers used for RTqPCR reactions exploring genes interest; Table S2: Antibodies used for cytometry applied to individualize cell population; Table S3: Purified and biotinylated antibodies used in ELISA.
