# Supplementary material for: Immunomodulatory Effects of High-Dose Irradiation Regimens in Renal Cell Carcinoma: Insights from an In Vitro Model with Human Peripheral Blood Mononuclear Cell
Source: Biomedicines. 2025 Aug 29;13(9):2107. doi: 10.3390/biomedicines13092107 (PMC12467837; doi:10.3390/biomedicines13092107)

biomedicines

MDPI

***Supplementary Table S1*:** References of primers used for RTqPCR reactions exploring genes interest.

Gene Name	Forward Primers	Reverse Primers
**β-Actine**	TAATGTCACGCACGATTTCCC	TCACCGAGCGCGGT
**GADPH**	ACCCACTCCTCCACCTTG	CTCTTGTGCTCTTGCTGGG
**HPRT**	CCCTGGCGTCGTGATTAG	ATGGCCTCCCATCTCCTT
**18S**	TCAAGAACGAAAGTCGGAGG	CTGGTCATGTTTTTTAGCCTCTTGT
**CD8**	TGGCCGCGCAGCTG	CTTGTTGGTTTGCACCTTTATGTATG
**CD4**	GGGAAATCAGGGCTCCTTCTTA	TGGTCCCAAAGGCTTCTTCTT
**CD11c**	AATTCAGGCGCACGTCAAA	ATCCCTACGGGCCCCATAT
**CD25**	GAGCTCAATCATCTGCTACGGA	TTGTCGTTGGAGGTGTTGTCTT
**FOXP-3**	TCACCTACGCCACGGTCA	CACAAAGCACTTGTGCAG
**CD80**	CCTCAATTTCTTTCAGCTCTTGGT	AGGACAGCGTTGCCACTTCT
**CD83**	GCCCTGCACAGCGTAAAGAAGA	TCTGTAGCCGTGCAAAACAAGTGA
**CD86**	GGGACTGAGTAACATTCTCTTTGTGA	GGCTTTGGTTTTGAGAGTTTGC
**Granzyme A**	ATTGCAAAAGACTGGGTGTTGAC	TGGCTCTTCCCTGGTTATTGA
**Granzyme B**	CGCCCCTACATGGCTTATCTT	CCCCCAAGGTGACATTTATGG
**INF-γ**	ATGTAGCGGATAATGGAACTC	GACATTCAAGTCAGTTACC
**TGF-β**	CGAGCCTGAGGCCGACTAC	CGGAGCTCTGATGTGTTGAAGA
**PD-1**	AGCCTGGAATTGCAGCTTCA	AAGTTGCATTCCAGGGTCACAT
**CTLA-4**	TTCTTCTCTTCATCCCTGTCTTCTG	GAGATGCATACTCACACACAAAGCT
**PDL-1**	GCTACAACTGGGCTGGCG	ATGTGTTGGAGAAGCTGCAGG
**IL-6**	CCAGGAGAAGATTCCAAAGATG	GGAAGGTTCAGGTTGTTTTCTG
**IL-8**	CTGGCCGTGGCTCTCTTG	TTCCACGTCAAAACGGTTCC
**HIF-1α**	GCCGCTGGAGACACAATCAT	CGTTTCAGCGGTGGGTAATG
**VEGF**	TGCCGCCACCACACCATCAC	GCCCTCCGGACCCAAAGTGC
**C-GAS**	GCGGTTTTGGAGAAGTTGAA	TGAATTCTGGGGACTTCCAG

**STNG**	ACTGTGGGGTGCCTGATAAC	TGGCAAACAAAGTCTGCAAG
**TREX-1**	GCATCTGTCAGTGGAGACCA	AGATCCTTGGTACCCCTGCT
**INF-β**	GAATGGGAGGCTTGAATACTGCCT	TAGCAAAGATGTTCTGGAGCATCTC

***Supplementary Table S2*:** Antibodies used for cytometry applied to individualize cell population

Antibody	Isotype
CD4 (VIT4) -VioBlue	Mouse IgG2a-VioBlue
CD8-VioGreen	Mouse IgG2a-VioGreen
CD3-PE-Vio770	Mouse IgG2a-PE-Vio770
CD335 (NKp46) -APC	Mouse IgG1-APC
CD11c-APC-Vio770	Mouse IgG2b-APC-Vio770
CD25-VioBright FITC	Mouse IgG2b-VioBright FITC
CD127-PE-Vio770	Mouse IgG2a-PE-Vio770
Anti-FoxP3-PE	Mouse IgG1-PE
CD14 -PE	Mouse IgG2-PE

***Supplementary Table S3*:** Purified and biotinylated antibodies used in ELISA.

	**purified antibodie**	**biotinylated antibodie**
Anti-TGF-β	Human IgG2a	Human IgG2a

Supplementary Figure S1:

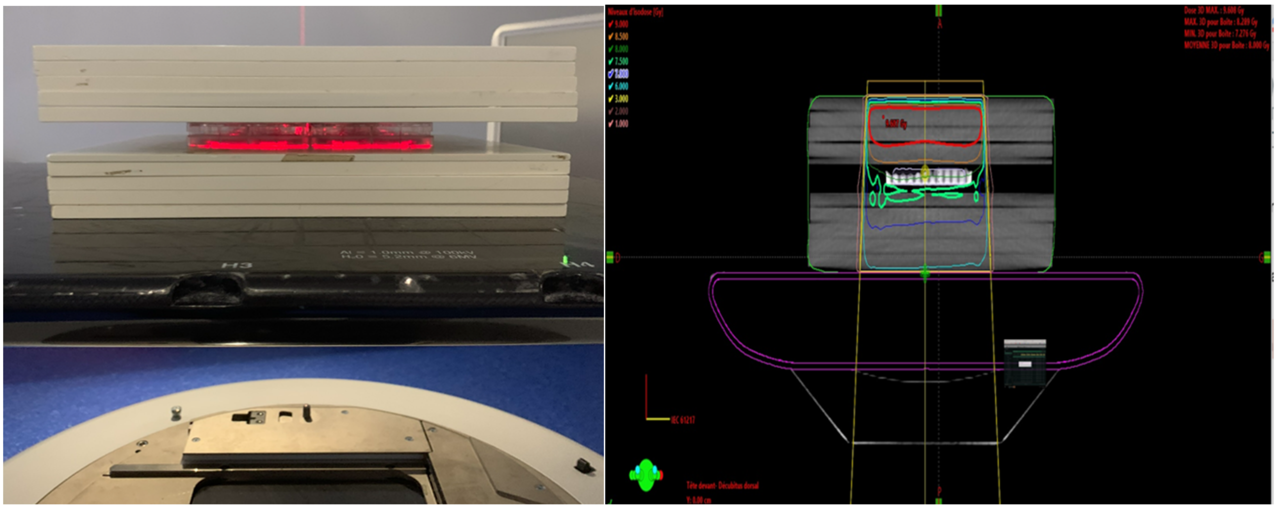

Figure S1. Overview of the dosimetry used for irradiation of ACHN & Caki-2 cells in-vitro. Doses of 1x8 or 3x8Gy with inter fraction interval of 24 hours were delivered to the containing well, using a single 6MV beam with a posterior-anterior direction.

Supplementary Figure S2: Experimental timeline

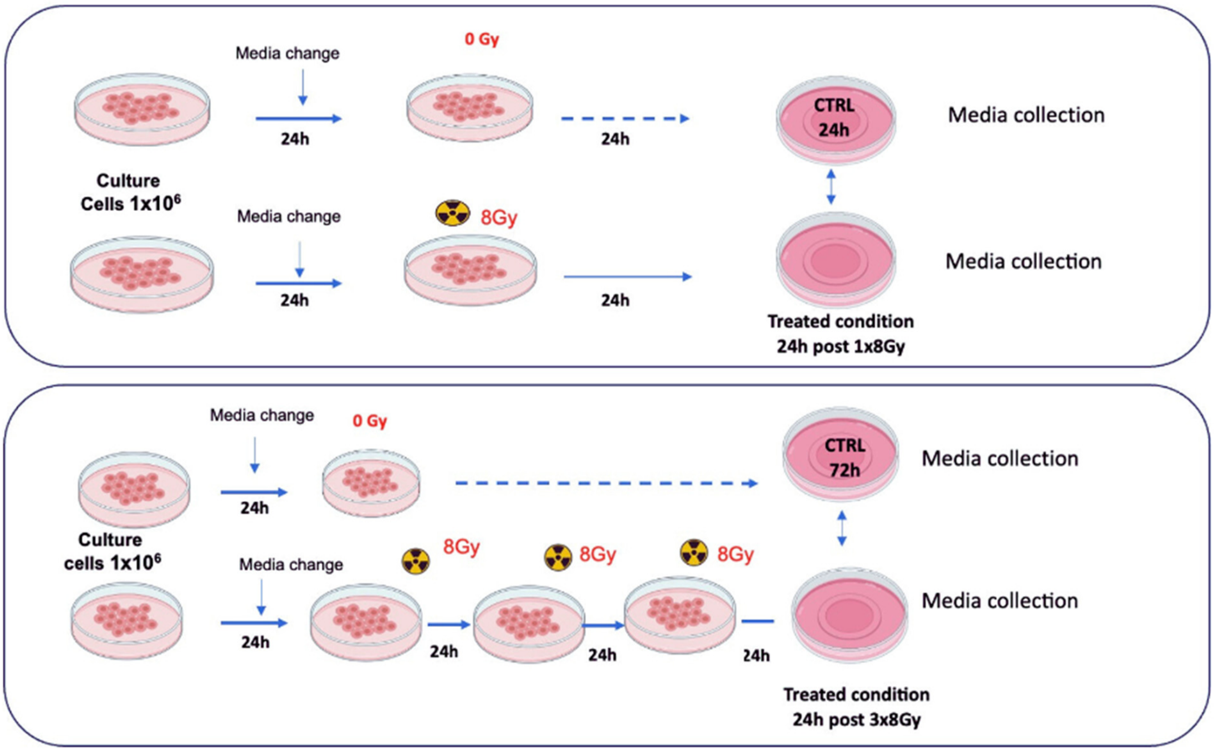

Supplementary Figure S3: strategy gating for characterizing the different subpopulations among PBMCs.

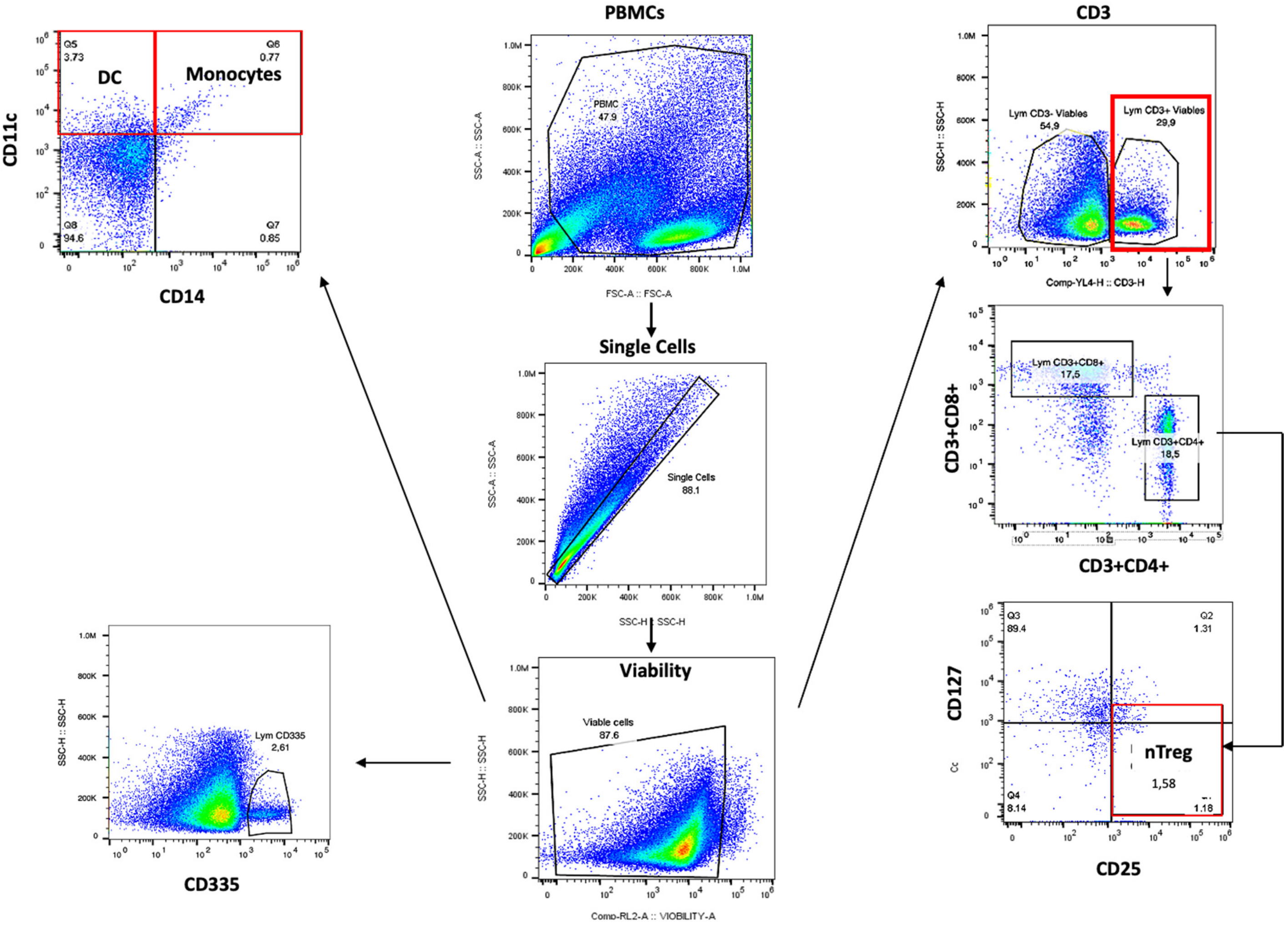

Supplement: Supplementary file 1 [file biomedicines-13-02107-s001.zip › biomedicines-3660866-supplementary.pdf]
